# Neighborhood conditions, diabetes, and risk of lower-body functional limitations among middle-aged African Americans: A cohort study

**DOI:** 10.1186/1471-2458-10-283

**Published:** 2010-05-27

**Authors:** Mario Schootman, Elena M Andresen, Fredric D Wolinsky, J Philip Miller, Yan Yan, Douglas K Miller

**Affiliations:** 1 Department of Medicine and Pediatrics, Washington University School of Medicine, St. Louis, MO, USA; 2 Department of Epidemiology and Biostatistics, College of Public Health and Health Professions, University of Florida, Gainesville, FL, USA; 3 Department of Health Management and Policy, College of Public Health, the University of Iowa, IA, USA; 4 Division of Biostatistics, Washington University School of Medicine, St. Louis, MO, USA; 5 Department of Surgery, Washington University School of Medicine, St. Louis, MO, USA; 6 Indiana University Center for Aging Research and Regenstrief Institute, Inc., Indiana University School of Medicine, Indianapolis, IN, USA

## Abstract

**Background:**

The relationship between presence of diabetes and adverse neighborhood and housing conditions and their effect on functional decline is unclear. We examined the association of adverse neighborhood (block face) and housing conditions with incidence of lower-body functional limitations among persons with and those without diabetes using a prospective population-based cohort study of 563 African Americans 49-65 years of age at their 2000-2001 baseline interviews.

**Methods:**

Participants were randomly sampled African Americans living in the St. Louis area (response rate: 76%). Physician-diagnosed diabetes was self reported at baseline interview. Lower-body functional limitations were self reported based on the Nagi physical performance scale at baseline and the three-year follow-up interviews. The external appearance of the block the respondent lived on and five housing conditions were rated by study interviewers. All analyses were done using propensity score methods to control for confounders.

**Results:**

109 (19.4%) of subjects experienced incident lower-body functional limitations at three-year follow-up. In adjusted analysis, persons with diabetes who lived on block faces rated as fair-poor on each of the five conditions had higher odds (7.79 [95% confidence interval: 1.36-37.55] to 144.6 [95% confidence interval: 4.45-775.53]) of developing lower-body functional limitations than the referent group of persons without diabetes who lived on block faces rated as good-excellent. At least 80 percent of incident lower-body functional limitations was attributable to the interaction between block face conditions and diabetes status.

**Conclusions:**

Adverse neighborhood conditions appear to exacerbate the detrimental effects on lower-body functioning associated with diabetes.

## Background

Diabetes has many adverse health effects, but one of the most important with serious consequences for living independently in the community is the impact of diabetes on lower-body function [1]. Poor lower-body function plays a crucial role in the disablement process and has been associated with increased disability days, physician contacts, fear of falling, falls, hip fracture, depression, nursing home placement, and mortality [2,3]. Not surprisingly, diabetes is a major risk factor for the development of lower-body-related functional limitations [4], and a substantial portion of population (adult) disability related to lower-extremity functioning is attributable to diabetes [5]. In the United States lower-body functional limitations are especially high among urban African Americans [6]. For these reasons, the issue of the effect of diabetes on lower-body functioning is particularly important for African Americans.

Neighborhood conditions have been shown to predict incident lower-body functional limitations [7,8] and adverse housing conditions have been associated with incident diabetes [9]. There are many potential pathways by which adverse neighborhood conditions might increase the risk of lower-body functional limitations, including increased stress, lower access to medical care, higher social isolation, and lower collective efficacy and social capital [10-12]. Based on the above-described associations, we hypothesized that adverse neighborhood and housing conditions may exacerbate the detrimental effects on lower-body functioning associated with diabetes. To our knowledge no studies have examined the potential interaction between diabetes and contextual factors such as neighborhood and housing conditions on the development of lower-body functional limitations. Adverse neighborhood and housing conditions may exacerbate the progression toward lower-body functional limitations among persons with diabetes as a result of complications (e.g., neuropathy, amputations), associated conditions (e.g., hypertension, obesity, stroke), low fruit and vegetable consumption and low physical activity, and reduced muscle strength [1], many of which are also associated with adverse neighborhood and housing conditions [13,14]. Therefore, we examined the association of adverse neighborhood (measured at the block face level) and housing conditions with incidence of lower-body dysfunction among persons with and those without diabetes using a longitudinal study of African Americans.

## Methods

### Baseline sample (wave 1)

The sampling design of the African American Health cohort has been described elsewhere [15]. Briefly, the African American Health study is a population-based cohort study of 998 noninstitutionalized African Americans recruited in 2000-2001 using multi-stage probability sampling. All subjects lived in one of two geographic sampling strata: either a poor, inner-city area (St. Louis), Missouri, United States) or more heterogeneous suburbs just northwest of the City of St. Louis. Interviewers (two thirds of whom were African American) with extensive study-specific training screened households for eligibility criteria, which involved self-reported black or African American race, birth date during 1936 through 1950, and Mini-mental Status Examination scores > 16. Subjects were paid volunteers. Sampling proportions were set to recruit approximately equal numbers of subjects from both areas (sampling strata). Each subject was weighted based on the selection probability and the response rate. When these weights are applied, the African American Health study sample represents the noninstitutionalized African American population in the two areas as of the 2000 Census.

All subjects received in-home, baseline evaluations (average = 2.5 hours) between September 2000 and July 2001. Baseline response was 76% (998/1320). All procedures were approved by the Institutional Review Boards of Saint Louis University and the University of Michigan.

### Follow-up sample (wave 4)

Follow-up in-home interviews averaging 1.5 hours were conducted 36 months after baseline assessments. Of the 998 persons who participated at baseline, 853 were successfully interviewed at follow-up. Since 51 persons had died between baseline and follow-up, the response for surviving subjects was 90.1% (853/947). No attrition bias during waves 1 through 4 was evident for any of the major variables involved in the current analysis. A total of 290 persons (weighted) had two or more lower body functional limitations at baseline and were excluded from further analysis. Thus, 563 persons (weighted) had one or fewer lower-body functional limitations at baseline and comprised the study sample.

### Lower-body functional limitations

Five items (0 = no difficulties to 1 = difficulty) from the Nagi physical performance scale assessed lower-body functional limitations, which were summed to form the outcome measure (ranging from 0 to 5) [6]. Items included difficulties in walking a quarter of a mile; walking up and down 10 steps without rest; standing for 2 hours; stooping, crouching, or kneeling; and lifting and carrying 10 pounds [16]. Subjects who expressed any difficulty or inability to perform the functional task at the time of the interview were considered to be limited in that task. Similar to other studies [7], we limited subjects in this study to those with one or fewer lower-body functional limitations at baseline in order to examine the risk of developing two or more lower-body functional limitations three years later. At follow-up, we defined incident lower-body functional limitations as reporting difficulty or being unable to perform at least two of the five physical tasks among those with one or fewer lower-body functional limitations at baseline.

### Adverse neighborhood and housing conditions

Assessment of neighborhood conditions was comprised of interviewer observations of the block face on which the respondent lived and participants' self-reported neighborhood desirability. An "objective" assessment of the external appearance of the block face (neighborhood) in front of the homes where the participants resided was done by the survey team using a previously published assessment tool [17] during household enumeration, which occurred an average of seven months before the participants were recruited and data were collected. Thus, block face conditions were collected independently from the interview data. Data about the housing conditions were collected at the time of the in-home interview and therefore not independently. On four-point scales (1 = excellent, 4 = poor) observers rated each of five characteristics: condition of houses, amount of noise (from traffic, industry, etc.), air quality, condition of the streets, and condition of the yards and sidewalks in front of homes where the participant lived. Weighted inter-rater Kappa statistics ranged from 0.58 (air quality) to 0.84 (condition of yards and sidewalks [18].

We also obtained a subjective measure of neighborhood conditions from respondents at baseline using a four-item scale of the neighborhood as a place to live, general feelings about the neighborhood, attachment to the neighborhood, and neighborhood safety from crime [19]. Participant responses were dichotomized for each condition, and the scale ranged from 0 to 4.

Assessment of housing conditions was an observed five-item scale based on the interviewer's ratings at the baseline interview of the cleanliness inside the building; physical condition of the interior; condition of furnishings; condition of the exterior of the building; and a global rating (all rated as excellent, good, fair, or poor). The test-retest reliability was at least 0.68 for each condition [9]. In the present analysis, each block face condition and each housing condition was dichotomized as either fair or poor versus good or excellent.

### Diabetes

The baseline interview asked respondents about the presence of physician-diagnosed diabetes (test-retest reliability in a subsample of African American Health study participants was 0.94) [20].

### Covariates

Baseline covariates included in the analysis were patterned after other African American Health cohort research [7]. Socio-demographic variables involved sampling stratum (inner city, suburb), age, gender, income categories, perceived income adequacy (having a comfortable income, having just enough to get by, not having enough to get by), educational attainment, marital status, employment status, number of persons in household, having health care insurance at the time of or during the 12 months prior to interview, and not being able to see a doctor because of cost during 12 months prior to interview. Social support was measured using five items from the Medical Outcomes Study social support instrument.

Health at baseline was measured by the self-rated health status question of the Short-form 36, depressive symptoms (score of at least 9 using the 11-item Center for Epidemiology Depressive Symptoms scale), a count of the number of self-reported physician-diagnosed severe chronic conditions ever experienced (asthma, chronic airway obstruction, heart failure, heart attack, angina, stroke, chronic kidney disease, arthritis, and cancer other than a minor skin cancer). Also assessed at baseline were body mass index, current smoking status, risk of alcohol abuse (CAGE), the Yale physical activity instrument, and grip strength.

**Figure 1:**
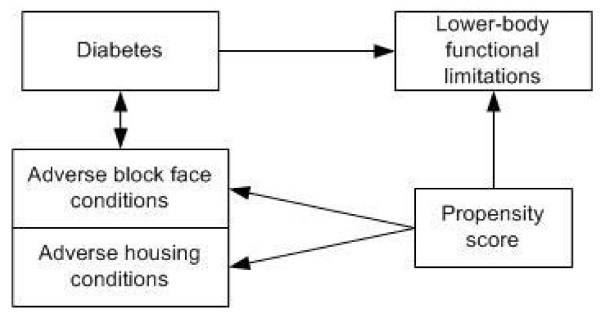
**Conceptual Model**.

The conceptual model of the relationship among the various types of variables is displayed in Figure 1. The association of interest is the relationship between diabetes and development of lower-body functional limitations as modified by adverse block face/housing conditions. Diabetes and the propensity score are predicting lower-body functional limitations. The association of diabetes with lower-body functional limitations is modified by adverse block face/housing conditions. The inclusion of the propensity score is aimed at estimating the unbiased association of interest.

### Statistical analysis

First, we calculated unadjusted measures of association (odds ratio [OR] and 95% confidence intervals [CI]) and interaction between diabetes and block face/housing conditions for the risk of incident lower-body functional limitation at 3-year follow-up. For each block face and housing condition, a single variable with four categories was created by combining a dichotomous neighborhood/housing variable (excellent/good vs. fair/poor) with the diabetes variable as part of the additive model [21]. Measures of interaction included the Interaction Contrast Ratio and the Attributable Proportion as measures of departure of additivity. The Interaction Contrast Ratio is the excess risk due to interaction relative to the risk without exposures [22]. If there is no superadditive interaction (null hypothesis), the Interaction Contrast Ratio equals 0. Interaction Contrast Ratio >0 indicates superadditivity, and Interaction Contrast Ratio < 0 indicates subadditivity. Odds ratios were substituted for the relative risks in the Interaction Contrast Ratio calculation. Attributable Proportion refers to the proportion of incident lower-body functional limitations attributable to the interaction among persons who experienced both exposures. If there is no interaction, the Attributable Proportion will equal 0.

To adjust for potential confounding from covariates, we used the propensity score method to obtain adjusted estimates of the measures of association and interaction between neighborhood/housing conditions with presence of diabetes on incident lower-body functional limitations [23,24]. All variables were included in the calculation of propensity scores. The propensity score is defined as the conditional probability of a person living under a certain neighborhood/housing condition and diabetes status, given covariates included. Multivariable logistic regression may be limited in its ability to control for confounders in studies when there are fewer than 10 events per variable analyzed [25]. The use of propensity scores has been proposed as an alternative that may be especially useful when multiple confounders are involved [26,27]. Propensity score methods produce estimates that are more accurate than logistic regression estimates when there are seven or fewer events per confounder, as was the case in the present study [24]. Similar to multivariable methods, unmeasured confounders are not included in the construction of propensity scores. Following previous work [28], we first estimated the probability in each of four categories of the interaction (diabetes Yes/No by block face/housing condition Fair/Poor vs. Good/Excellent) for each individual using a nominal multinomial logistic regression model. Next, we assigned a weight using the inversed predicted probability that the individual was observed in one of the categories. The propensity score-adjusted estimates incorporated these weights in the analyses. Then, we obtained the interaction indices using the log odds ratio estimates from the logistic regression model. Also, we examined the interaction indices for hypertension and arthritis with the contextual variable to challenge the robustness of the findings.

Confidence intervals for all effect measures were calculated using the bootstrap percentile methodology. For a 95% confidence interval the 2.5^th ^and 97.5^th ^percentiles of the empirical distribution of Interaction Contrast Ratios were calculated from 1,000 data sets resampled with replacement from the original data. All statistical analyses were conducted using SAS software, version 9.1.

## Results

Of 563 subjects with zero or one lower-body functional limitations at baseline, 109 (19.4%) experienced two or more lower-body functional limitations at the 3-year follow-up. Eighteen percent of subjects reported having diabetes at baseline and 82 percent did not. Of those with diabetes, the percentage of subjects who lived on one of the ten block face or housing conditions rated as fair or poor ranged from 10.3% to 23.3%. Of those without diabetes, the percentage of subjects who lived on one of the ten block face or housing conditions rated as fair or poor ranged from 17.2% to 20.2%. There were no statistical differences in block face or housing conditions between persons with and those without diabetes. Baseline characteristics of the study population and factors associated with incident lower-body functional limitations in univariate analysis have been described briefly in Table 1 and more extensively elsewhere [7]. Briefly, persons who were older, unable to visit a doctor because of the cost, scored nine or more on the Center for Epidemiology Depressive Symptoms 11-item scale, experienced greater number of severe chronic conditions, or had one lower-body functional limitations at baseline were more likely to experience incident lower-body functional limitations at 3-year follow-up. Persons were less likely to have incident lower-body functional limitations at follow-up when they had lived more than five years at the present address or were overweight at baseline.

**Table 1:** Prevalence of selected characteristics at baseline and unadjusted risk of three-year incident lower body functional limitation for subjects in the African-American Health study

		Unadjusted risk of incident lower body functional limitation at 3-year follow-up	
	Baseline measure** (weighted n = 563)	**Odds ratio**	**95% CI**

Age (mean[s.d])	56.1 (4.7)	1.06	1.01 - 1.11
Gender			
Women vs. Men	54.6%	1.46	0.95 - 2.24
Length of time at present address			
More than 5 yrs vs. Less than 5 years	73.1%	0.83	0.34 - 0.82
Objective income			
< $20,000 vs. > = $50,000	17.4%	1.68	0.95 - 2.94
$20,000 - < $50,000 vs. > = $50,000	48.8%	1.32	0.29 - 6.07
Highest level of education			
< 12 years vs. 12 years or more	21.0%	0.74	0.43 - 1.27
Unable to visit doctor because of cost			
Yes vs. No	6.4%	2.35	1.14 - 4.83
Center for Epidemiology Depressive Symptoms 11-item score ≥9			
Yes vs. No	12.5%	1.89	1.08 - 3.32
No. of severe chronic conditions (per condition)	0.8 (1.0)	1.56	1.28 - 1.90
Lower body limitation at baseline			
One vs. None	29.2%	3.56	2.30 - 5.49
Body Mass Index			
>= 30.0 vs. < 25.0	35.5%	0.61	0.36 - 1.04
25.0 - 29.9 vs. < 25.0	40.7%	0.48	0.28 - 0.81

* CI: confidence interval, s.d.: standard deviation.

** Mean (SD), unless noted.

### Incidence

Of 563 subjects with zero or one lower-body functional limitations at baseline, the percentage that developed two or more lower-body functional limitations at 3-year follow-up varied according to the participant's diabetes status and each of the five block-face conditions. For example, 65% percent of persons with diabetes who lived on block faces with yards and sidewalks in fair-poor condition developed lower-body functional limitations. In contrast, 19.4 percent of persons without diabetes who lived on block faces with yards and sidewalks in fair-poor condition developed lower-body functional limitations. About 17 percent of persons who lived on block faces with good-excellent conditions (regardless of diabetes status) developed lower-body functional limitations. Similar results were observed for the other four block-face conditions. Little difference was present examining each of the five housing conditions.

### Interaction between block face conditions and diabetes status

In unadjusted analysis, (a) persons with diabetes residing on block faces with good-excellent conditions and (b) persons without diabetes living on block faces with fair-poor conditions generally were not significantly more likely to develop lower-body functional limitations three years later than the referent group of persons without diabetes who lived on block faces rated as good or excellent (additional File 1). The only exceptions were for the rating of air quality for both groups and street and road quality for the second group, for which the odds ratios were 2.0 to 2.4. In contrast, persons with diabetes who lived on block faces rated as fair or poor on each of the five conditions had seven to 14 higher odds of developing lower-body functional limitations than the referent group. An interaction existed between block face condition and presence of diabetes for each of the five conditions (all Interaction Contrast Ratio >1.0). At least 75 percent of the incidence of lower-body functional limitations was attributable to this interaction for each of the block face conditions (additional File 1).

In adjusted analyses, we observed parameter estimates that generally were larger than those in the unadjusted analysis for all conditions (additional File 2). The Attributable Proportion of the incidence of lower-body functional limitations due to the interaction involving the other four conditions was at least 80 percent. The values of the Interaction Contrast Ratios indicate that the excess risk due to the interaction was large relative to the risk without either exposure.

### Interaction between housing conditions and diabetes status

In unadjusted analysis, persons with diabetes who lived under housing conditions rated as good or excellent and those rated as fair or poor had about two times higher odds of developing lower-body functional limitations at 3-year follow-up compared to the reference group (additional File 3). However, based on the Interaction Contrast Ratio and the Attributable Proportion there was no interaction between housing condition and diabetes. In adjusted analysis, there was also no interaction between any of the housing conditions and diabetes, and the associations with the two types of exposures on their own became insignificant (additional File 4).

Sensitivity analyses were performed with hypertension and arthritis to examine the specificity of the interaction between diabetes and the contextual variables. However, no interaction was found for hypertension and arthritis (data not shown), suggesting the observed interaction is specific to diabetes and neighborhood conditions.

## Discussion and Conclusions

This study has demonstrated the importance of the interaction of diabetes and neighborhood conditions acting in concert on the deterioration of lower-body functional limitations in an urban African American population. This "double jeopardy" suggests that both risk factors combined increased the risk of lower-body functional limitations considerably more than diabetes or adverse block face conditions alone [29]. In contrast, we found no evidence of an interaction between diabetes and housing conditions on incident lower-body functional limitations. To our knowledge, ours is the first study that shows a powerful synergy between neighbor conditions and presence of disease on subsequent adverse functional outcome.

Adverse neighborhood conditions may exacerbate the progression toward lower-body dysfunction among persons with diabetes as a result of diabetic complications (e.g., heart disease, visual impairment, neuropathy, ulceration, lower extremity amputation), associated conditions (e.g., obesity, hypertension), poor diet and low physical activity, and reduced muscle strength [1]. We adjusted for these risk factors by including them in the propensity score, except for lower-extremity disorders such as peripheral neuropathy, foot ulcers, peripheral arterial disease, and lower-extremity amputation, for which we had little data. These lower-extremity conditions have been associated with numbness in the extremities, and trouble with gait and balance, and lower-body functional limitations [30]. It is possible that adverse neighborhood conditions accelerate the decline toward lower-body functional limitations through lower-extremity conditions in persons with diabetes. However, discerning the meditational pathways was not the purpose of our study. In efforts to prevent future lower-body dysfunction among persons with diabetes by intervening upon lower-extremity disorders, attention to environmental circumstances (especially block face conditions) and the individual's interaction with them needs to be part of the interventional strategy. The findings appear robust with respect to sensitivity analysis.

Study limitations include a study sample that involves a single race, a single city, and a restricted age range, all of which may limit generalizability. However, focusing on a single race allows the disentanglement of race and socioeconomic status. Notably, African Americans experience more diabetes and more diabetic complications than does the majority population [31], so the effect of the diabetes-neighborhood interaction may be particularly strong in African Americans. We used self-reported diabetes to classify cases of diabetes and thus some cases of prevalent diabetes probably were missed. Despite the very high test-retest reliability of self-reported diabetes in the African American Health data [20], misclassification of diabetes status may still be present, which could lead to biased results. However, unless misclassification of self-reported diabetes was dependent upon block face condition, our results would likely be a conservative estimate of the true relationships. Another limitation is that we had in some instances only 24 persons with diabetes who lived in fair or poor block face conditions at baseline. The relatively small size of this group resulted in wide confidence intervals in some results, but unity was never included.

Finally, in most studies of neighborhood effects, multiple study participants are nested within their neighborhood, requiring the use of multilevel statistical techniques. In this study sample, there were 551 block faces, 363 on which only one participant resided (65.9%). Only 3.6 percent of block faces contained five or more participants. We were not able to use multilevel statistical techniques because there was not enough clustering of participants within block faces to support a robust multi-level analytic approach. In a previous study, we randomly selected one subject per block face from the block faces with more than 1 subject and showed that parameter estimates were very similar to our findings using propensity scores [7].

In summary, there appears to be a powerful interaction between adverse block face conditions and the presence of diabetes on decline is a crucial factor for maintaining health and independence (i.e., lower-body physical functioning) in urban-dwelling middle-aged African Americans. Further research is needed to investigate the mediators of this powerful interaction.

## Competing interests

The authors declare that they have no competing interests.

## Authors' contributions

MS conceived of the study and drafted the manuscript, EMA, FDW, JPM participated in drafting of the manuscript, YY performed the statistical analysis, DKM helped conceive of the study and participated in drafting of the manuscript. All authors read and approved the final manuscript.

## Pre-publication history

The pre-publication history for this paper can be accessed here:

http://www.biomedcentral.com/1471-2458/10/283/prepub

## Supplementary Material

Additional file 1 Unadjusted measures of association (odds ratio and 95% confidence intervals) and interaction (interaction contrast ratio and attributable proportion and 95% confidence intervals) between diabetes and block face conditions for the risk of incident lower-body functional limitation at 3-year follow-up (weighted n = 563). Click here for file

Additional file 2 Propensity score adjusted measures of association (odds ratio and 95% confidence intervals) and interaction (interaction contrast ratio and attributable proportion and 95% confidence intervals) between diabetes and block face conditions for the risk of incident lower-body functional limitation at 3-year follow-up (weighted n = 563).* Click here for file

Additional file 3 Unadjusted measures of association (odds ratio and 95% confidence intervals) and interaction (interaction contrast ratio and attributable proportion and 95% confidence intervals) between diabetes and housing conditions for the risk of incident lower-body functional limitation at 3-year follow-up (weighted n = 563). Click here for file

Additional file 4 Propensity score adjusted measures of association (odds ratio and 95% confidence intervals) and interaction (interaction contrast ratio and attributable proportion and 95% confidence intervals) between diabetes and housing conditions for the risk of incident lower-body functional limitation at 3-year follow-up (weighted n = 563). Click here for file
