# Supplementary material for: Neighborhood conditions, diabetes, and risk of lower-body functional limitations among middle-aged African Americans: A cohort study
Source: BMC Public Health. 2010 May 27;10:283. doi: 10.1186/1471-2458-10-283 (PMC2885338; doi:10.1186/1471-2458-10-283)
Supplement: Additional file 1 — Unadjusted measures of association (odds ratio and 95% confidence intervals) and interaction (interaction contrast ratio and attributable proportion and 95% confidence intervals) between diabetes and block face conditions for the risk of incident lower-body functional limitation at 3-year follow-up (weighted n = 563). [file 1471-2458-10-283-S1.DOC]

Additional file 1. Unadjusted measures of association (odds ratio and 95% confidence intervals) and interaction (interaction contrast ratio and attributable proportion and 95% confidence intervals) between diabetes and block face conditions for the risk of incident lower-body functional limitation at 3-year follow-up (weighted n=563).

|  | Good-excellent block  face conditions | | | | | | | | | | | Fair-poor block  face conditions | | | | | | | | | | | | |  | | | | | | | | |
| --- | --- | --- | --- | --- | --- | --- | --- | --- | --- | --- | --- | --- | --- | --- | --- | --- | --- | --- | --- | --- | --- | --- | --- | --- | --- | --- | --- | --- | --- | --- | --- | --- | --- |
|  | |  | | Without  diabetes | | | | | With diabetes | | | | | | Without diabetes | | | | | With diabetes | | | | | |  | | | | | | | |
| Type of condition | | | Prevalence (%) of fair/poor quality | | | OR | OR | | | 95% CI | | | OR | | | 95% CI | | OR | | | 95% CI | | Interaction  contrast  ratio | | | | | 95% CI | | Attributable proportion | | 95% CI | |
| Housing conditions | | | 24.7 | | 1.00* | | | 1.42 | | | 0.67, 2.97 | | | 1.37 | | | 0.75, 2.61 | | 7.20 | | | 3.98, 12.88 | | 5.28 | | | 2.57, 10.44 | | 0.75 | | 0.52, 0.90 | |  |
| Noise | | | 21.0 | | 1.00* | | | 1.48 | | | 0.76, 3.04 | | | 1.39 | | | 0.76, 2.61 | | 7.91 | | | 4.64, 14.52 | | 5.92 | | | 2.93, 5.92 | | 0.76 | | 0.52, 0.89 | |  |
| Air quality | | | 18.3 | | 1.00* | | | 2.01 | | | 1.09, 3.75 | | | 2.37 | | | 1.30, 4.67 | | 13.82 | | | 6.74, 38.17 | | 10.15 | | | 3.65, 10.15 | | 0.75 | | 0.47, 0.92 | |  |
| Street & road quality | | | 20.9 | | 1.00* | | | 1.54 | | | 0.78, 3.12 | | | 2.35 | | | 1.17, 4.56 | | 14.19 | | | 7.37, 30.56 | | 10.99 | | | 5.12, 25.99 | | 0.79 | | 0.60, 0.91 | |  |
| Yard & sidewalk quality | | | 24.6 | | 1.00* | | | 1.08 | | | 0.53, 2.34 | | | 1.20 | | | 0.70, 2.21 | | 9.52 | | | 5.34, 17.73 | | 8.12 | | | 4.40, 15.49 | | 0.86 | | 0.69, 0.95 | |  |

*Referent odds ratio
