# Supplementary material for: Neighborhood conditions, diabetes, and risk of lower-body functional limitations among middle-aged African Americans: A cohort study
Source: BMC Public Health. 2010 May 27;10:283. doi: 10.1186/1471-2458-10-283 (PMC2885338; doi:10.1186/1471-2458-10-283)
Supplement: Additional file 2 — Propensity score adjusted measures of association (odds ratio and 95% confidence intervals) and interaction (interaction contrast ratio and attributable proportion and 95% confidence intervals) between diabetes and block face conditions for the risk of incident lower-body functional limitation at 3-year follow-up (weighted n = 563).* [file 1471-2458-10-283-S2.DOC]

Additional file 2. Propensity score adjusted measures of association (odds ratio and 95% confidence intervals) and interaction (interaction contrast ratio and attributable proportion and 95% confidence intervals) between diabetes and block face conditions for the risk of incident lower-body functional limitation at 3-year follow-up (weighted n=563).*

| Good-excellent block  face conditions | | | | | | Fair-poor block  face conditions | | | | | |  | | | | |
| --- | --- | --- | --- | --- | --- | --- | --- | --- | --- | --- | --- | --- | --- | --- | --- | --- |
|  | Without diabetes | | With diabetes | | | Without diabetes | | | With diabetes | | |  | | | | |
| Type of condition | | OR | | OR | 95% CI | | OR | 95% CI | | OR | 95% CI | | Interaction contrast ratio | 95% CI | Attributable proportion | 95% CI |
| Housing conditions | | 1.00** | | 1.15 | 0.57, 2.22 | | 1.46 | 0.70, 3.05 | | 7.79 | 1.36, 37.55 | | 6.01 | -0.46, 35.22 | 0.80 | -0.33, 0.96 |
| Noise | | 1.00** | | 1.13 | 0.56, 2.30 | | 1.74 | 0.87, 3.47 | | 144.60 | 4.45, 775.53 | | 143.0 | 2.64, 772.79 | 0.99 | 0.52, 1.00 |
| Air quality | | 1.00** | | 1.53 | 0.68, 3.14 | | 2.84 | 1.34, 6.17 | | 23.65 | 3.73, 93.68 | | 19.62 | 0.00, 89.27 | 0.86 | 0.00, 0.97 |
| Street & road quality | | 1.00** | | 1.14 | 0.56, 2.39 | | 3.39 | 1.24, 8.00 | | 79.53 | 5.85, 347.67 | | 76.18 | 2.18, 345.68 | 0.95 | 0.36, 0.99 |
| Yard & sidewalk quality | | 1.00** | | 1.01 | 0.53, 2.00 | | 1.39 | 0.64, 3.02 | | 20.79 | 2.64, 82.37 | | 19.09 | 1.04, 80.45 | 0.93 | 0.33, 0.99 |

* Variables included in the propensity score: sampling stratum, age, gender, income, perceived income adequacy, educational attainment, marital status, employment status, number of persons in household, health care insurance, not being able to see a doctor because of cost, social support, self-rated health status, depressive symptoms, a count of the number of chronic conditions, body mass index, risk of alcohol abuse, and physical activity.

**Referent odds ratio
