# Supplementary material for: Neighborhood conditions, diabetes, and risk of lower-body functional limitations among middle-aged African Americans: A cohort study
Source: BMC Public Health. 2010 May 27;10:283. doi: 10.1186/1471-2458-10-283 (PMC2885338; doi:10.1186/1471-2458-10-283)
Supplement: Additional file 3 — Unadjusted measures of association (odds ratio and 95% confidence intervals) and interaction (interaction contrast ratio and attributable proportion and 95% confidence intervals) between diabetes and housing conditions for the risk of incident lower-body functional limitation at 3-year follow-up (weighted n = 563). [file 1471-2458-10-283-S3.DOC]

Additional File 3. Unadjusted measures of association (odds ratio and 95% confidence intervals) and interaction (interaction contrast ratio and attributable proportion and 95% confidence intervals) between diabetes and housing conditions for the risk of incident lower-body functional limitation at 3-year follow-up (weighted n=563).

|  | Good-excellent housing  conditions | | | | | | | | | | | Fair-poor housing conditions | | | | | | | | | | | | |  | | | | | | | | |
| --- | --- | --- | --- | --- | --- | --- | --- | --- | --- | --- | --- | --- | --- | --- | --- | --- | --- | --- | --- | --- | --- | --- | --- | --- | --- | --- | --- | --- | --- | --- | --- | --- | --- |
|  | |  | | Without  diabetes | | | | | With diabetes | | | | | | Without diabetes | | | | | With diabetes | | | | | |  | | | | | | | |
| Type of condition | | | Prevalence (%) of fair/poor quality | | | OR | OR | | | 95% CI | | | OR | | | 95% CI | | OR | | | 95% CI | | Interaction  contrast  ratio | | | | | 95% CI | | Attributable proportion | | 95% CI | |
| Cleanliness inside building | | | 21.1 | | 1.00* | | | 2.04 | | | 1.14, 3.92 | | | 0.98 | | | 0.50, 2.16 | | 1.94 | | | 1.01, 4.14 | | -0.06 | | | -2.10, 1.66 | | -0.04 | | -1.37, 0.56 | |  |
| Physical conditions interior | | | 19.5 | | 1.00* | | | 2.07 | | | 1.13, 3.81 | | | 1.02 | | | 0.45, 2.26 | | 2.00 | | | 1.03, 3.93 | | -0.06 | | | -2.30, 1.57 | | -0.03 | | -1.44, 0.56 | |  |
| Furnishings | | | 22.6 | | 1.00* | | | 2.12 | | | 1.15, 4.11 | | | 1.54 | | | 0.75, 3.06 | | 2.88 | | | 1.36, 5.69 | | 0.12 | | | -2.36, 2.44 | | 0.04 | | -1.13, 0.57 | |  |
| Outside condition | | | 20.6 | | 1.00* | | | 2.09 | | | 1.12, 3.92 | | | 1.20 | | | 0.62, 2.40 | | 2.32 | | | 1.06, 4.66 | | 0.05 | | | -2.14, 1.85 | | 0.02 | | -1.14, 0.55 | |  |
| Overall condition | | | 19.6 | | 1.00* | | | 1.98 | | | 1.05, 3.72 | | | 1.30 | | | 0.70, 2.63 | | 3.04 | | | 1.40, 6.72 | | 0.72 | | | -1.73, 3.69 | | 0.26 | | -0.90, 0.70 | |  |

*Referent odds ratio
