# Supplementary material for: Neighborhood conditions, diabetes, and risk of lower-body functional limitations among middle-aged African Americans: A cohort study
Source: BMC Public Health. 2010 May 27;10:283. doi: 10.1186/1471-2458-10-283 (PMC2885338; doi:10.1186/1471-2458-10-283)
Supplement: Additional file 4 — Propensity score adjusted measures of association (odds ratio and 95% confidence intervals) and interaction (interaction contrast ratio and attributable proportion and 95% confidence intervals) between diabetes and housing conditions for the risk of incident lower-body functional limitation at 3-year follow-up (weighted n = 563). [file 1471-2458-10-283-S4.DOC]

Additional File 4. Propensity score adjusted measures of association (odds ratio and 95% confidence intervals) and interaction (interaction contrast ratio and attributable proportion and 95% confidence intervals) between diabetes and housing conditions for the risk of incident lower-body functional limitation at 3-year follow-up (weighted n=563).

| Good-excellent housing  conditions | | | | | | Fair-poor housing  conditions | | | | | | |  | | | |
| --- | --- | --- | --- | --- | --- | --- | --- | --- | --- | --- | --- | --- | --- | --- | --- | --- |
|  | Without diabetes | | With diabetes | | | Without diabetes | | | | With diabetes | | |  | | | |
| Type of condition | | OR | | OR | 95% CI | | OR | 95% CI | OR | | 95% CI | Interaction contrast ratio | | 95% CI | Attributable proportion | 95% CI |
| Cleanliness inside building | | 1.00** | | 1.39 | 0.71, 2.87 | | 0.85 | 0.36, 2.14 | 0.59 | | 0.07, 4.92 | -0.57 | | -2.65, 3.63 | -1.08 | -18.5, 0.82 |
| Physical conditions interior | | 1.00** | | 1.32 | 0.65, 2.47 | | 1.27 | 0.36, 3.22 | 0.64 | | 0.08, 5.76 | -0.76 | | -3.31, 4.23 | -1.33 | -22.93, 0.82 |
| Furnishings | | 1.00** | | 1.36 | 0.67, 2.77 | | 1.35 | 0.47, 3.75 | 1.38 | | 0.27, 4.51 | -0.31 | | -3.23, 2.61 | -0.23 | -5.84, 0.76 |
| Outside condition | | 1.00** | | 1.28 | 0.59, 2.75 | | 1.28 | 0.54, 2.91 | 0.49 | | 0.08, 5.43 | -0.95 | | -3.52, 3.42 | -2.06 | -20.74, 0.79 |
| Overall condition | | 1.00** | | 1.24 | 0.62, 2.49 | | 1.59 | 0.66, 3.47 | 2.46 | | 0.27, 11.67 | 0.67 | | -2.35, 9.76 | 0.28 | -6.59, 0.89 |

* Variables included in the propensity score: sampling stratum, age, gender, income, perceived income adequacy, educational attainment, marital status, employment status, number of persons in household, health care insurance, not being able to see a doctor because of cost, social support, self-rated health status, depressive symptoms, a count of the number of chronic conditions, body mass index, risk of alcohol abuse, and physical activity

**Referent odds ratio
